# Expression differences in *Aphidius ervi* (Hymenoptera: Braconidae) females reared on different aphid host species

**DOI:** 10.7717/peerj.3640

**Published:** 2017-08-21

**Authors:** Gabriel I. Ballesteros, Jürgen Gadau, Fabrice Legeai, Angelica Gonzalez-Gonzalez, Blas Lavandero, Jean-Christophe Simon, Christian C. Figueroa

**Affiliations:** 1 Instituto de Ciencias Biológicas, Universidad de Talca, Talca, Chile; 2 Millennium Nucleus Centre in Molecular Ecology and Evolutionary Applications in the Agroecosystems, Universidad de Talca, Talca, Chile; 3 School of Life Sciences, Arizona State University, Tempe, AZ, United States of America; 4 Institute for Evolution and Biodiversity, Westfälische Wilhelms-Universität Münster, Münster, Germany; 5 GenScale, INRIA Centre Rennes, Rennes, France; 6 Institute of Genetics, Environment and Plant Protection, INRA, Le Rheu, France

**Keywords:** Phenotypic plasticity, Local adaptation, Parasitoid wasps, Transcriptome, Aphid pest control

## Abstract

The molecular mechanisms that allow generalist parasitoids to exploit many, often very distinct hosts are practically unknown. The wasp *Aphidius ervi,* a generalist koinobiont parasitoid of aphids, was introduced from Europe into Chile in the late 1970s to control agriculturally important aphid species. A recent study showed significant differences in host preference and host acceptance (infectivity) depending on the host *A. ervi* were reared on. In contrast, no genetic differentiation between *A. ervi* populations parasitizing different aphid species and aphids of the same species reared on different host plants was found in Chile. Additionally, the same study did not find any fitness effects in *A. ervi* if offspring were reared on a different host as their mothers. Here, we determined the effect of aphid host species (*Sitobion avenae* versus *Acyrthosiphon pisum* reared on two different host plants alfalfa and pea) on the transcriptome of adult *A. ervi* females. We found a large number of differentially expressed genes (between host species: head: 2,765; body: 1,216; within the same aphid host species reared on different host plants: alfalfa versus pea: head 593; body 222). As expected, the transcriptomes from parasitoids reared on the same host species (pea aphid) but originating from different host plants (pea versus alfalfa) were more similar to each other than the transcriptomes of parasitoids reared on a different aphid host and host plant (head: 648 and 1,524 transcripts; body: 566 and 428 transcripts). We found several differentially expressed odorant binding proteins and olfactory receptor proteins in particular, when we compared parasitoids from different host species. Additionally, we found differentially expressed genes involved in neuronal growth and development as well as signaling pathways. These results point towards a significant rewiring of the transcriptome of *A. ervi* depending on aphid-plant complex where parasitoids develop, even if different biotypes of a certain aphid host species (*A. pisum*) are reared on the same host plant. This difference seems to persist even after the different wasp populations were reared on the same aphid host in the laboratory for more than 50 generations. This indicates that either the imprinting process is very persistent or there is enough genetic/allelic variation between *A. ervi* populations. The role of distinct molecular mechanisms is discussed in terms of the formation of host fidelity.

## Introduction

Parasitoids are widely used in biological control programs, which are based on introduced, naturalized, natural or released parasitoid wasps (Starý et al., 1993). Most parasitoids used for controlling aphids are endoparasitoids, which lay eggs inside their host as part of their life cycle and eventually killing it. Endoparasitoids may attack many related host species (e.g., same family) (Loxdale & Harvey, 2016). However, they have at the same time the problem to overcome or avoid different defense mechanisms of their varied host species in order to survive through adulthood (Jones et al., 2015). One way to do this is the formation of host races, i.e., populations that are adapted to a specific host (Stireman et al., 2006). Alternatively, the parasite may change its “phenotype” depending on the host it encounters and parasitizes, i.e., it shows adaptive phenotypic plasticity (Crispo, 2008). A third alternative is that several host species can be parasitized by the parasitoid using the same strategy/phenotype. The former two mechanisms will lead to differences in host preference and host acceptance, affecting behavioral traits associated to host selection (Zepeda-Paulo et al., 2013) but should not necessarily affect or change parasitoid fitness (Rivero, 2000; Wang et al., 2016).

One of the most widely used species in biological control programs is *Aphidius ervi,* a worldwide distributed koinobiont endoparasitoid of several Macrosiphinae aphid species such as the pea aphid *Acyrthosiphon pisum* (Henry et al., 2010; Stilmant et al., 2008) and the grain aphid *Sitobion avenae* (Cameron, Powell & Loxdale, 1984). The parasitoid *A. ervi* was introduced into Chile from Europe in the late 70’s as part of an aphid biological control program in cereals. The introduced *A. ervi* wasps successfully parasitized both *A. pisum* on legumes (e.g., alfalfa) and *S. avenae* on cereals (e.g., wheat) (Starý, 1993; Zepeda-Paulo et al., 2013) although these two aphids differ in several important aspects (e.g., host range usage, body size and colour, semiochemicals present in the cuticle, cornicular secretions, defensive reactions, etc.) (Daza-Bustamante, Fuentes-Contreras & Niemeyer, 2003). The recent introduction of a small number of individuals and natural occurrence of *A. ervi* attacking different aphid species opens interesting questions regarding the molecular basis and evolution of host preference and host specific adaptations (host races). Adaptations to different hosts have been described and confirmed as a plausible speciation mechanism (ecological speciation; Abrahamson & Blair, 2008; Schluter, 2000). Interestingly, whereas differences in host preference and acceptance have been described previously from parasitoids reared or collected from different aphid host species, no fitness effects were detected if those parasitoids were forced to lay their eggs in suitable hosts they were not reared on (Zepeda-Paulo et al., 2013). These results suggest that the preference for the natal host (i.e., host fidelity) is not under direct selection and those parasitoids may show adaptive phenotypic plasticity (Daza-Bustamante et al., 2002; Zepeda-Paulo et al., 2013). Indeed, no host race specific differentiation has been detected in Chilean *A. ervi* populations (Zepeda-Paulo et al., 2013) and a high gene flow predominantly mediated by male dispersion was found between populations (Zepeda-Paulo et al., 2015). Therefore, phenotypic plasticity should be playing a key role in the observed host fidelity (Henry, Roitberg & Gillespie, 2008; Zepeda-Paulo et al., 2013). However, it is unclear how much phenotypic plasticity the parasitoid needs to exploit different hosts or whether they can use identical mechanisms and strategies when parasitizing different hosts. One way to test between these options is to compare the transcriptomes of *A. ervi* females reared on different hosts. If *A. ervi* uses the same strategy for parasitizing both hosts their transcriptome should be very similar.

Despite its widespread use in applied and fundamental research, no genomic or transcriptomic information are available for *A. ervi* (Colinet et al., 2014). This study uses RNAseq to *de novo* assemble and annotate the first representative transcriptome for *A. ervi*. Additionally, our experimental setup allowed us to identify differentially expressed genes between *A. ervi* parasitizing two different host species (*Acyrthosiphon pisum* and *Sitobion avenae*) for two different body parts (head and body) and the same host species (*Acyrthosiphon pisum*) reared on two different host plants (alfalfa and pea). This will allow us to determine how much phenotypic plasticity at the transcriptome level *A. ervi* shows and whether it can use the same strategy when it parasitizes different hosts.

## Materials and Methods

### Insect collection and rearing

**Figure 1:**
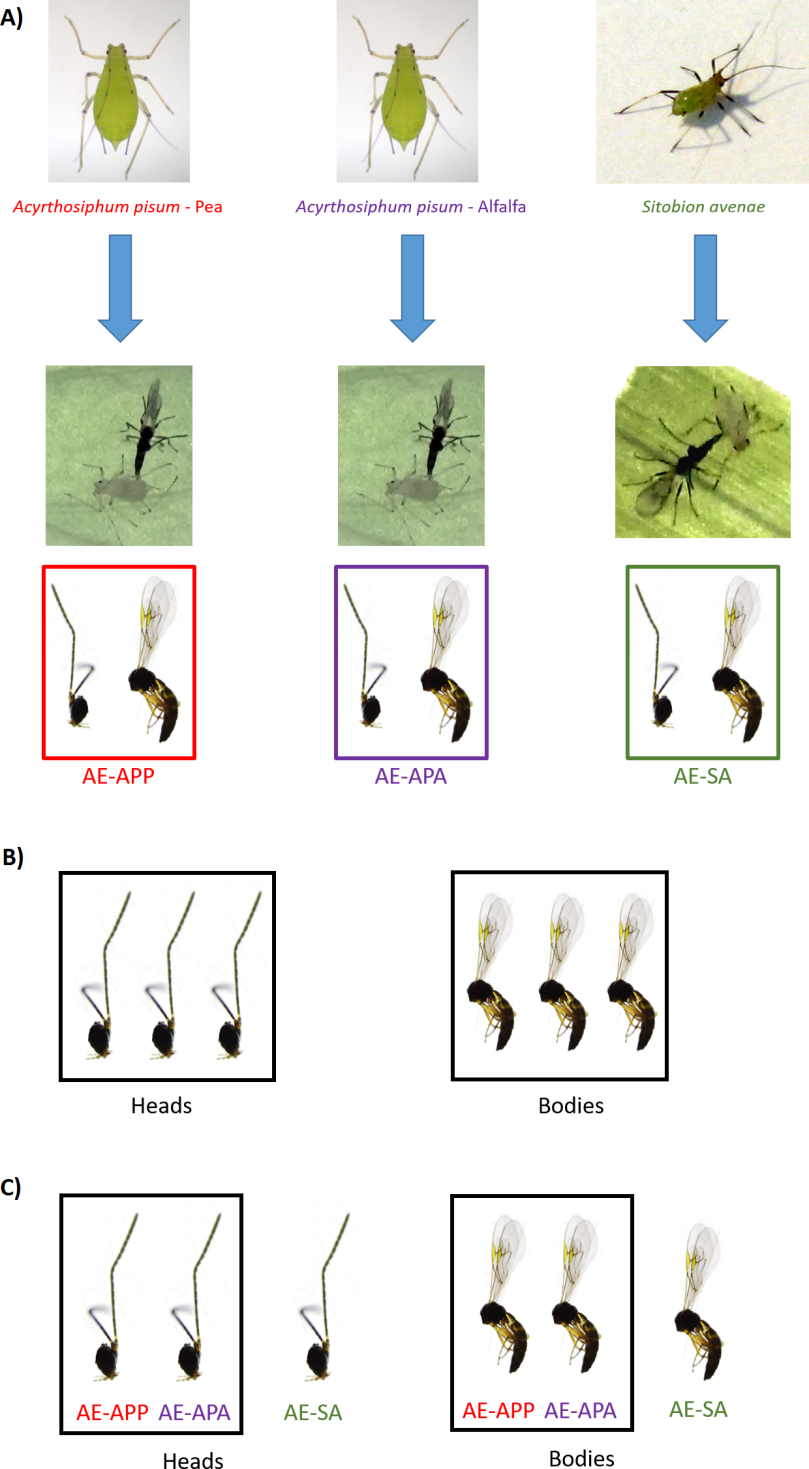
Sampling design for RNA sequencing and differential expression analysis in the aphid parasitoid wasp *Aphidius ervi*. (A) Parasitoid population rearing. (B) DE analysis between heads and bodies (three libraries/tissue). (C) DE analysis between parasitoid host races.

Parasitized aphids (two host races of *Acyrthosiphon pisum* and *Sitobion avenae*) were collected from fields of legumes and cereals in two different geographic zones in Chile: Region de Los Rios (S39°51′, W73°7′) and Region del Maule (S35°24′, W71°40′). The alfalfa race of *A. pisum* (APA) was sampled on alfalfa (*Medicago sativa* L.) and the pea race (APP) was sampled on pea (*Pisum sativum L.*) (Peccoud et al., 2008), while the grain aphid *Sitobion avenae* (SA) was sampled on wheat (*Triticum aestivum* L.). Pea aphids (from both alfalfa and pea races) were maintained in the laboratory on broad bean (*Vicia faba* L.) while grain aphids were maintained on barley (*Hordeum vulgare* L.). Both host plants have been used previously for aphid and parasitoid rearing in other studies (Sepúlveda et al., 2017b; Zepeda-Paulo et al., 2013). Parasitized aphids were reared under laboratory conditions that allowed continuous reproduction (20°C, D16/N8 photoperiod) (Zepeda-Paulo et al., 2013). *Aphidius ervi* parasitoids were collected as larvae from parasitized aphids, recognizable as mummies and kept separated in vials until adult parasitoids emerged. Species and sex of each emerging parasitoid was determined using a standard taxonomic key (Starý, 1995). In order to establish inbred populations, a single, isolated naive *A. ervi* virgin female was mated with a naive virgin male for 24 h in a petri dish with diluted honey and water for sustenance. Mated females were then offered new aphid hosts *ad libitum* in a separate cage. These inbred lineages were propagated for approximately 60 generations before samples were taken for the RNAseq experiments. All *A. ervi* parasitoids were reared on the same host aphid species from which they were originally collected (further on called natal host). Thus, three different and highly inbred *A. ervi* laboratory populations were established: (i) *A. ervi* population originally collected from *A. pisum* living on alfalfa (Ae-APA) (ii) *A. ervi* population originally collected from *A. pisum* living on pea (Ae-APP) and (iii) *A. ervi* population originally collected from *S. avenae* living on wheat (Ae-SA) (Fig. 1). Each week, new aphid infested plants were introduced into the *A. ervi* rearing cages for parasitoid population maintenance, together with vials containing diluted honey and water for adult parasitoid feeding. All aphid populations were free of known secondary endosymbionts; their presence was evaluated using the amplification of specific 16S rDNA from whole-body aphid DNA based on the set of known primers described by Peccoud et al. (2014). This method allows screening of different symbionts, including the protective bacteria *Hamiltonella defensa* and *Regiella insecticola*, and also *Serratia symbiotica*, *Rickettsia, Rickettsiella* and *Spiroplasma* (Sepúlveda et al., 2017a).

### RNA collection and sequencing

Adult female parasitoids were collected alive from each one of the three caged parasitoid populations (*N* = 20 per population) (Fig. 1) and stored in 1.5 ml centrifuge tubes containing RNALater (QIAGEN, Hilden, Germany) at −20°C until dissection and RNA extraction. Heads and bodies were dissected on ice using a sterile scalpel and pooled in six different samples. For each sample, total RNA was extracted using the RNEasy Plant Mini Kit (QIAGEN, Hilden, Germany) following the manufacturer’s instructions and eluted in 50 µl of RNAse free water. Total RNA was quantified by spectrophotometry (Epoch Microplate Spectrophotometer, Biotek) and fluorometry (Qubit 3.0, Qubit RNA Broad Range Assay Kit), and integrity checked in a Bioanalyzer 2100 RNA Nano Kit (Agilent, Santa Clara, CA, USA). Recovered total RNA was precipitated using 0.1 volumes of Sodium Acetate 3 M and 2 volumes of 100% Ethanol and shipped to Macrogen Korea for library preparation and sequencing. Ribosomal RNA was depleted from total RNA using the Ribo-Zero rRNA Removal Kit for enrichment of both insect mRNA and non poly-adenilated mRNA that might be present in *A. ervi* sequenced samples. Remaining RNA was used for library construction using TruSeq Stranded Total RNA Sample Preparation Kit (Illumina, San Diego, CA, USA), tagged, pooled and sequenced using an Illumina HiSeq 2000 (2 ×100 bp, Paired End libraries; Macrogen, Seoul, South Korea). Raw transcriptome data was deposited in NCBI’s Sequence Read Archive database under BioProject ID: PRJNA377544.

### Illumina sequence processing and transcriptome assembly

Illumina RNA-seq libraries were quality checked with FastQC ver. 0.11.3 (http://www.bioinformatics.babraham.ac.uk/projects/fastqc) in order to assess the presence of adapters derived from sequencing, overrepresented kmers, read length and overall read quality scores. All libraries were processed with Trimmomatic ver. 0.35 (Bolger, Lohse & Usadel, 2014) to remove any remaining TruSeq adapter sequence and to eliminate low quality bases (*Q* < 3) from reads. After sequence processing, all remaining sequences shorter than 36 bp long were also removed from all datasets. Clean Illumina datasets were pooled *in silico* by concatenating library files. Before assembly, ribosomal RNA reads were removed by mapping the libraries using Bowtie ver. 1.1.1 (Langmead et al., 2009) against a custom rRNA database created from insect ribosomal sequences downloaded from NCBI and keeping non-mapped reads. The remaining high quality reads were *de novo* assembled with Trinity ver. 2.0.6 using default parameters. Metrics for *de novo* assembly were obtained with QUAST ver. 2.3 (number of contigs, total length, N50, largest contig, %GC, etc.) (Gurevich et al., 2013) while transcriptome completeness was assessed by benchmarking the assembled transcriptome using BUSCO (benchmarking universal single-copy orthologs) v.1.1b1 (Simão et al., 2015). To determine whether this transcriptome encodes for one or more set of core genes conserved across a range of Arthropod species, a “completeness score” was calculated (Moreton, Izquierdo & Emes, 2016). A total of 2,675 near-universal single-copy orthologs from Arthropod species were used as reference core genes (available at busco.ezlab.org; Simão et al., 2015). Additionally, reference protein sequences of *A. ervi* were downloaded from both the Non Redundant (NR) and Transcriptome Shotgun Assembly (TSA) sequence databases available at NCBI (*N* = 422; Colinet et al., 2014). These sequences were used as a custom reference protein database for BLASTx alignments using the assembled transcriptome as query.

### Annotation and functional gene classification

Homology searches of contigs from the assembled *de novo* transcriptome were performed locally with BLASTX using the NR database (NCBI) as reference (April 2016 version), setting an *e*-value of 1^*e*−5^ as threshold. Any contig that showed homology with at least one gene or protein was designated as a hit contig, while contigs with no hits were disregarded for any of the follow up analysis. Contigs with top-hit to non-insect species (e.g., prokaryote, yeast, vertebrates, etc.) were removed from the assembly and stored separately for future analysis because we were focused only on insect genes. Insect species reference lists available in NCBI Taxonomy browser were used to identify contigs with top-hits for insects (search criteria “insecta”, 200 levels displayed). To further improve the accuracy of differentially expressed genes, all contigs aligning to the same protein were grouped using BLASTx homology results and were sorted by alignment BitScore. The sequence with the highest BitScore was considered as the best blast hit, selected and designed as an annotated contig (Ono et al., 2015). The insect-filtered, non-redundant contig fasta dataset was loaded into Blast2GO ver. 2.8 (Conesa & Götz, 2008) altogether with BLASTx results in XML format. We also performed InterPro annotation, Gene Ontology (GO) term assignment, enzyme code and pathway annotation using Kyoto Encyclopedia of Genes and Genomes (KEGG) term integrated into Blast2GO. Successfully annotated transcripts were categorized and assigned to GO terms from different GO categories (molecular function, cellular component and biological process). The final contig annotation table was obtained from the combination of “Top-Blast table” and “sequence table”, both exported from Blast2GO.

### Differential gene expression analysis

The insect filtered, non-redundant reference transcriptome was used as a basis for differential gene expression studies between tissues (separate for head and body) (Fig. 1). Note: each individual sample was based on 20 individual females. Additionally, differential expression studies were carried out in order to detect and describe unique expressed transcripts for parasitoid lines from different aphid hosts (Ae-APP = *A. pisum*—Pea; Ae-APA = *A. pisum*—Alfalfa; Ae-SA = *S. avenae*) (Fig. 1). Gene abundance estimation was performed by separately aligning the libraries to the reduced reference transcriptome using the align_and_estimate script included in Trinity (ver. 2.0.6). This script automated the reference transcript, performed library read alignment to the reference using Bowtie2 (ver. 2.2.4; Langmead et al., 2009), and estimated read abundances from mapping results per library with the RSEM package included in Trinity (ver. 2.0.6.; Li & Dewey, 2011, and it was used to interpret and analyze Bowtie2 mapping results. RSEM was used to combine each count matrix and to build up a raw transcript expression matrix and a TMM-normalized expression matrix (script abundance_estimates_to_matrix.pl); this raw counts matrix was further used for Differential expression (DE) analysis at tissue level (heads vs. bodies, three libraries per sample), aphid host species and host rearing plant species. DE analysis was performed with edgeR Bioconductor package implemented in R using the provided run_DE_analysis script in Trinity ver. 2.0.6. The package edgeR was selected as it has a relatively high sensitivity and specificity in DE analysis of pooled samples compared to other methods of analysis. Genes that had at least 4-fold change values with a FDR-corrected *p*-value of 0.01 or lower were considered as significantly differentially expressed between libraries/tissues. The annotation of DE contigs was performed by combining TMM-normalized expression profiles for each contig with the annotated transcriptome tables generated in Blast2GO together with BLASTx results. Uniquely, differentially expressed contigs were detected for all libraries. In the case of the SA lineage (both for heads and bodies), a contig was considered as overexpressed only if its expression profile was 4-fold times higher when compared to both APA and APP populations. For both *A. pisum* races, contigs were considered as overexpressed only if their fold-change was at least 4-fold times higher (FDR-corrected *p*-value < 0.01) compared to the SA race in both APA and APP populations. The GO term enrichment was performed using Fisher’s exact test in Blast2GO.

## Results

### *Aphidius ervi* reference transcriptome assembly

We generated a *de novo* transcriptome for *A. ervi* using transcriptomic datasets obtained from the sequencing of six Illumina libraries (NCBI SRA accession PRJNA377544). These datasets were obtained from pooled female adult parasitoids reared from different aphid hosts (APP = *A. pisum*—Pea; APA = *A. pisum*—Alfalfa; SA = *S. avenae*) (Table 1). All transcripts were concatenated to generate a reference transcriptome library that could act as further reference for other studies involving *A. ervi* (GenBank accession GFLW01000000). The RNAseq generated 431,237,778 raw reads (2 ×  100 bp, paired end libraries). After pre-processing and removal of ribosomal RNA reads, 237,214,294 reads remained. Using Trinity, filtered reads were assembled into 135,659 contigs (N50 of 1,516 bp, mean length of 675.95 bp). Among the assembled contigs, 38,567 were less than 300 bp (28.4%), 75,458 contigs were between 301 bp and 1,000 bp (55.6%), while 21,634 contigs had a size over 1,000 bp (16%). The assembly completeness was also evaluated using BUSCO (benchmarking universal single-copy orthologs) showing that 76.6% complete conserved genes were found in our assembly, 13.7% corresponded to fragmented conserved genes while only 9.8% of single-copy ortholog genes were missing. Our results are similar to BUSCO metrics reported for other *de novo* insect transcriptomes assemblies such as the Western tarnished plant bug *Lygus hesperus* assembly performed with Illumina datasets (Tassone et al., 2016). Additionally, all previously known *A. ervi* sequences (*N* = 422) retrieved from NCBI were present in our transcriptomic assembly.

**Table 1:** Summary of *Aphidius ervi* transcriptomic libraries and assembly statistics.

Sequencing		
Library sequenced	Raw reads	Filtered Reads
Ae-APA	122,819,778	115,025,660
Ae-APP	124,329,988	113,456,324
Ae-SA	184,088,012	161,916,086
Total	431,237,778	390,398,070
Minus rRNA		237,214,294

Assembly			
	Reference *de novo*	Blast Hits	Insect only
Total transcript number	135,676	33,853	17,763
Total length	91,710,298	47,294,638	25,440,092
longest transcript	19,479	19,479	19,479
N50	1,516	2,027	1,983
%GC	30.15%	31.02%	30.67%

### Sequence annotation

**Figure 2:**
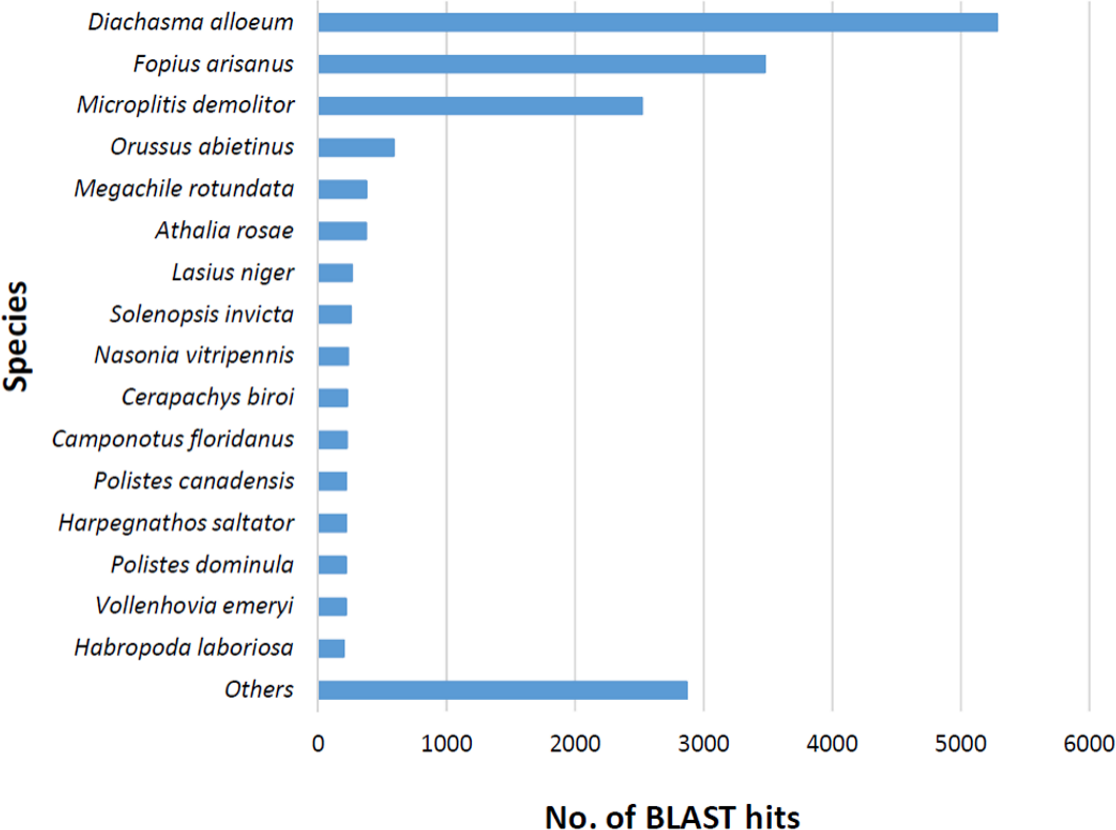
Species distribution of unigene sequences of the aphid parasitoid wasp *Aphidius ervi* transcripts to other insect species using homologous BLASTx hits and NR-NCBI database.

**Figure 3:**
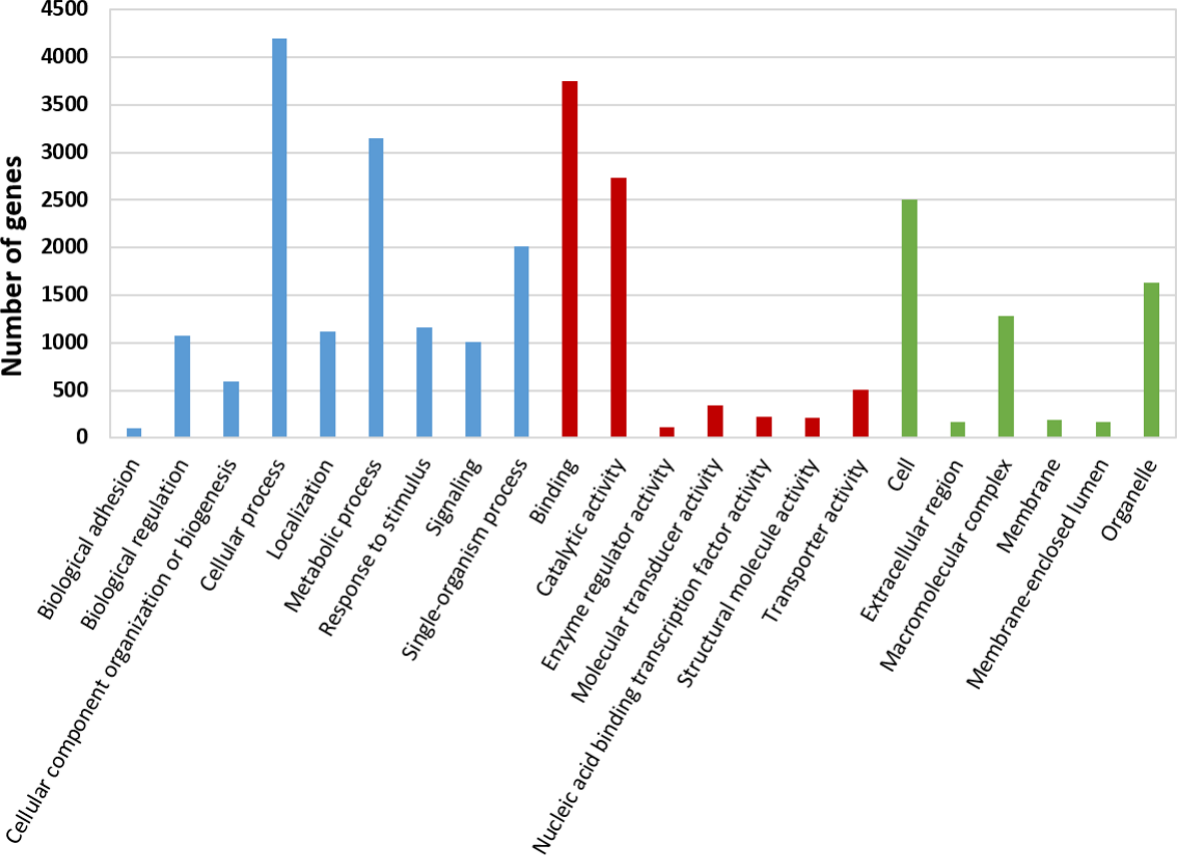
Gene Ontology (GO) annotations for the reference transcriptome of *Aphidius ervi* separated by GO categories (Biological process: blue. Molecular function: red. Cellular component: green).

The BLASTx alignments revealed that 33,853 contigs were annotated to a known protein within the NR database (24.9% of total contigs) (Table 1). Most transcript sequences with protein hits matched to other braconid endoparasitoids such as *Diachasma alloeum* (parasitoid of the apple maggot *Rhagoletis pomonella*), *Fopius arisanus* (parasitoid of Tephritid fruit flies) and *Microplitis demolitor* (parasitoid of noctuid larvae) (Fig. 2), all species for which *de novo* transcriptomes have been published (Burke & Strand, 2012; Calla et al., 2015). This dataset was further filtered to remove redundant contigs by using best-blast hit criteria (see ‘Materials and Methods’). Additionally, annotated sequences from non-insect organisms were removed; both steps filtered out 16,090 contigs. The remaining 17,763 contigs were the basis for all follow up analyses (annotation and differential expression). A total of 10,492 contigs (59.1%) could be annotated based on their sequence homology with GO terms. As contigs can be assigned to more than one GO category, 14,614 contigs were assigned to biological process, 7,945 contigs were classified under molecular function, and 5,976 were classified in cellular component (Fig. 3). This GO term distribution is congruent with other insect transcriptomes already sequenced (Hwang et  al., 2016).

### Transcriptomic differences between tissues and function of transcripts with different expression levels

**Figure 4:**
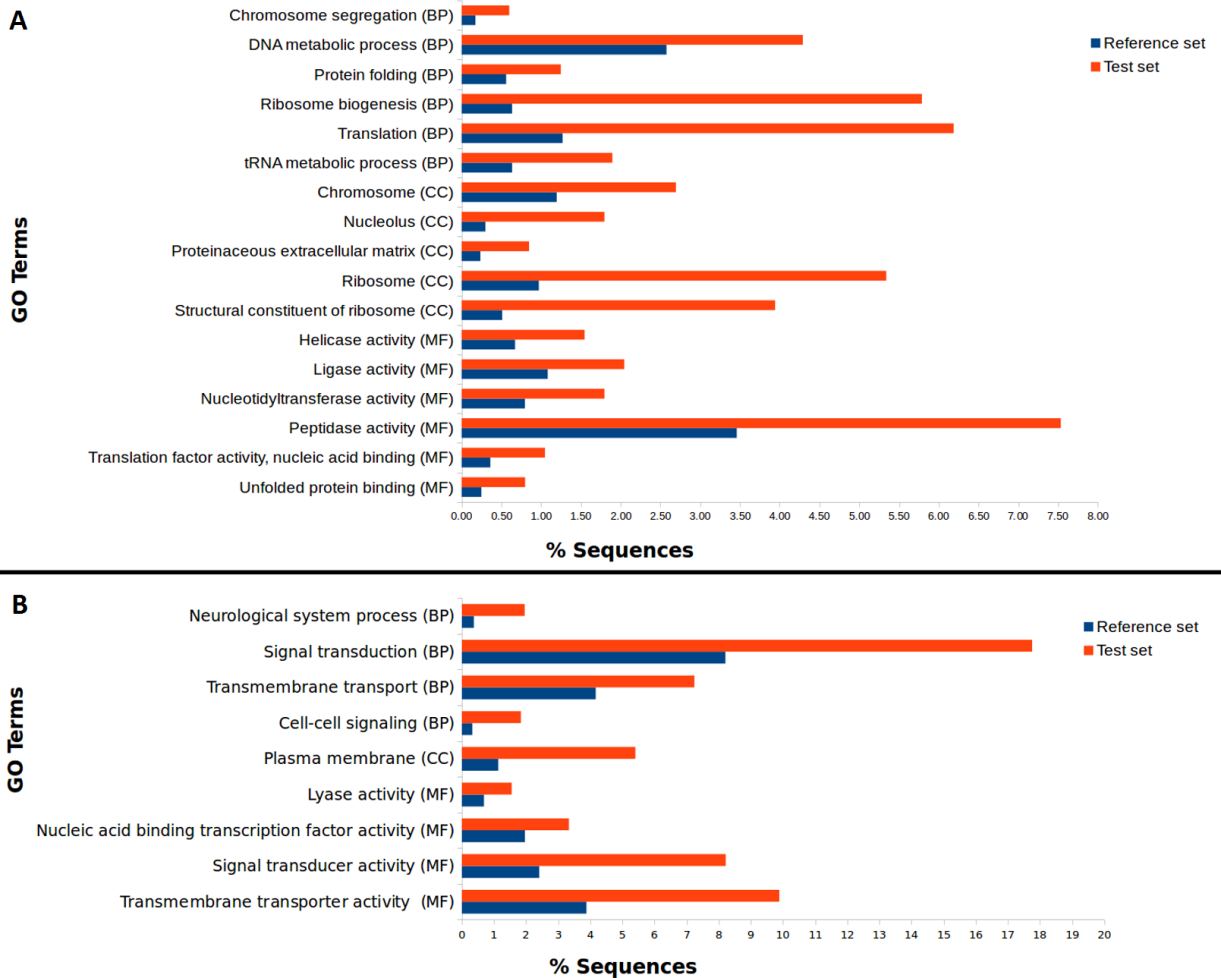
Differential GO term distribution between *Aphidius ervi* bodies and heads (Blast2GO Fisher’s exact test with FDR correction). Reference set: full *A. ervi* transcriptome. Test set: % of sequences associated to GO-enriched terms. (A) enriched GO-terms in bodies. (B) enriched GO-terms in heads. CC, Cellular Component; MF, Molecular Function; BP, Biological Process.

We used our filtered transcriptome as a reference dataset to perform Differential Expression (DE) analysis for *A. ervi* reared on the three different hosts. Our results indicate that there is a good correlation within head samples and within body samples, while clear differential expression patterns are observed for different body parts (Fig. 4; Fig. S1). Differential expression analysis showed that 6,389 transcripts are being differentially expressed between head and body samples (*N* = 3,445 up-regulated in heads, *N* = 2,944 up-regulated in bodies). The GO Term enrichment analysis was performed between differentially expressed genes and indicated the most enriched GO terms for bodies and heads (Fig. 4). “Signal transduction” and “signal transducer activity” were among the most enriched GO terms for heads, hence indicating that signaling and response stimulus associated transcripts are prevalent in the head transcriptomes, which is expected as RNA was extracted from whole heads including the brain and chemical sense organs such as antennae (Glaser et al., 2015). In the case of bodies, “ribosome biogenesis” and “peptidase activity”, were, amongst others, the most enriched GO terms found. As we extracted RNA from headless bodies without further dissection, the enriched “peptidase activity” could probably reference to venom proteins such as serine proteases or gamma-glutamyl transpeptidases, which have also been described previously for *A. ervi* (Colinet et al., 2014). These venom proteins are injected into the host at oviposition and would have a role in the modulation of the aphid physiology by inducing apoptosis of host ovaries and arresting host reproduction (Colinet et al., 2014; Falabella et al., 2007).

### Transcriptomic differences between parasitoid lines and function of transcripts with different expression levels

**Figure 5:**
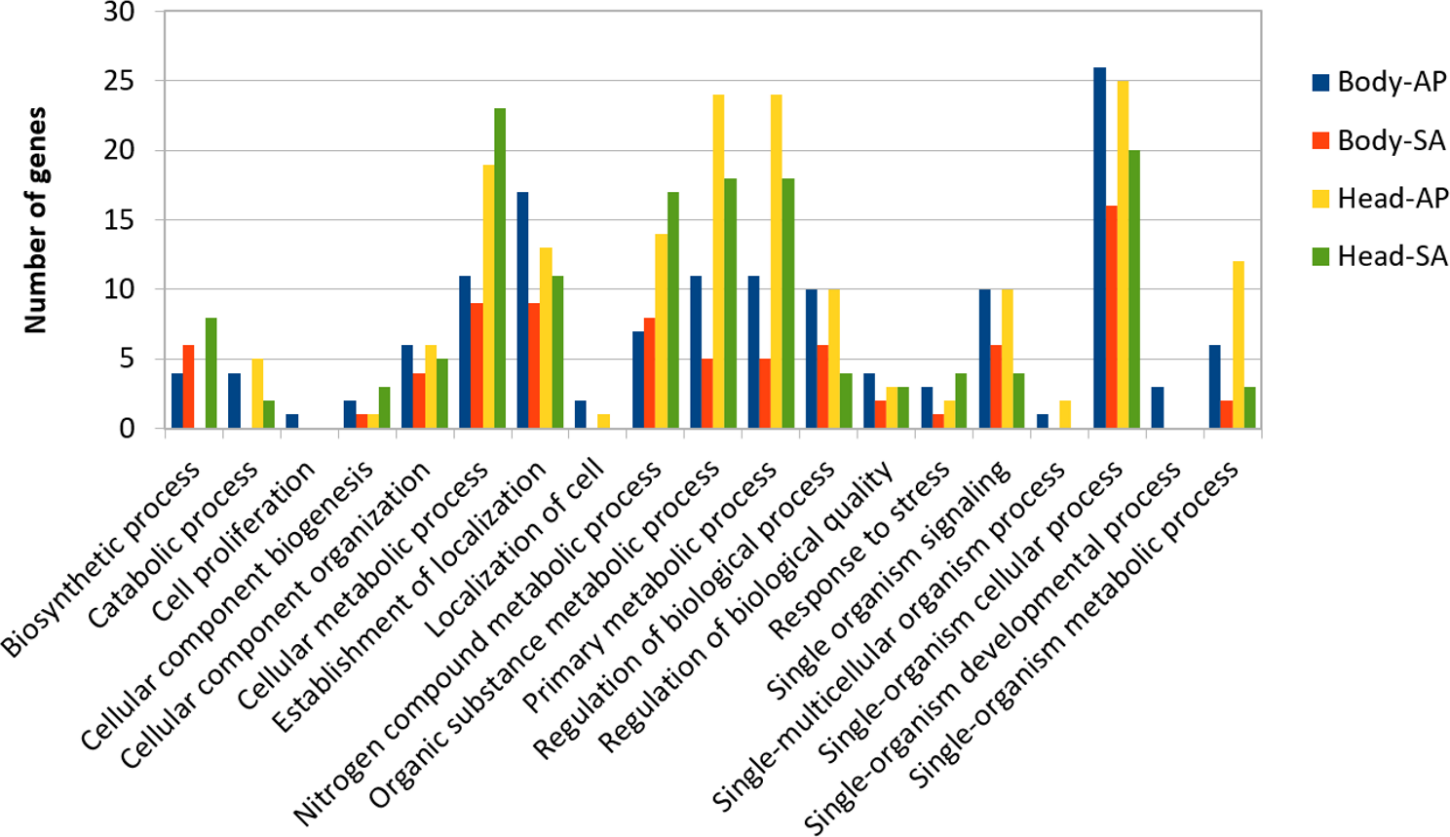
GO term distribution—biological process for genes with different expression patterns between *Aphidius ervi* libraries.

**Figure 6:**
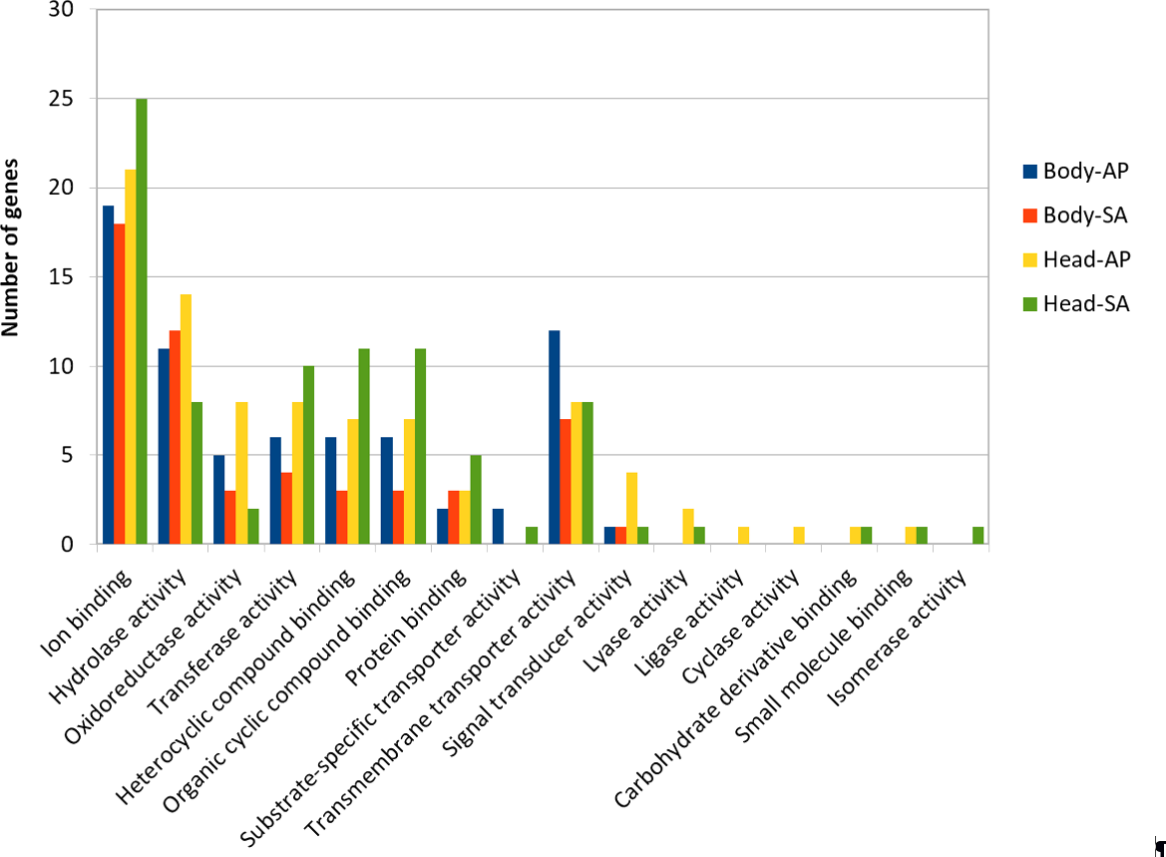
GO term distribution—Molecular function for genes with different expression patterns between *Aphidius ervi* libraries.

At body level, we found 239 transcripts with differential expression patterns between *A. ervi* populations that were originally collected from *A. pisum* on alfalfa and pea, respectively (149 up-regulated genes for Ae-AP and 90 up-regulated genes in Ae-SA; *q-value* < 0.01, fold-change >  4), while at head level 390 transcripts showed differential expression (219 upregulated in Ae-AP and 171 upregulated in Ae-SA). All differentially expressed transcripts were annotated using the results from BlastX alignments while GO terms were assigned using Blast2GO mapping results, which in turn was used to identify the functions of genes displaying different expression patterns between populations and tissues (Figs. 5 and 6); this approach was used because GO term enrichment analysis failed to find any enriched term. Top 20 differentially expressed gene lists (ranked first according to fold-change) are reported in Table 2 (Complete lists in File S1).

**Table 2:** Top 20 genes with differential expression patterns between *Aphidius ervi*-AP and *A. ervi*-SA on two different tissues (heads and bodies).

ID	Library	Sequence description	Log2-fold change	*P*-Value	FDR-adjusted *p*-value
TR52610-c0_g1_i1	Ae-AP Body	sodium hydrogen exchanger 7 isoform x4	13.57	6.48E–20	3.73E–16
TR27559-c2_g1_i1	Ae-AP Body	rho gtpase-activating protein 190 isoform x1	13.27	1.18E–19	4.82E–16
TR36885-c0_g2_i1	Ae-AP Body	oxidoreductase glyr1 homolog	12.17	2.86E–16	3.71E–13
TR42270-c3_g5_i10	Ae-AP Body	calcium-activated potassium channel slowpoke	12.12	3.43E–16	6.80E–13
TR27536-c4_g1_i1	Ae-AP Body	disco-interacting protein 2 isoform x1	11.84	3.76E–10	7.65E–08
TR42293-c6_g1_i8	Ae-AP Body	lon protease mitochondrial isoform x1	11.44	3.38E–14	2.23E–11
TR52097-c0_g1_i3	Ae-AP Body	synaptojanin-1 isoform x1	11.14	2.84E–13	1.85E–10
TR30823-c0_g1_i18	Ae-AP Body	zinc finger protein rotund isoform x3	10.98	3.93E–12	9.37E–10
TR55124-c1_g1_i1	Ae-AP Body	ryanodine receptor isoform x6	10.95	2.18E–11	6.15E–09
TR55070-c8_g1_i6	Ae-AP Body	dynamin isoform x2	10.91	3.11E–12	7.91E–10
TR4006-c7_g2_i9	Ae-AP Body	down syndrome cell adhesion molecule-like protein dscam2 isoform x30	10.88	1.15E–12	4.56E–10
TR13038-c0_g3_i4	Ae-AP Body	excitatory amino acid transporter isoform x1	10.84	3.07E–12	8.35E–10
TR42270-c3_g5_i5	Ae-AP Body	calcium-activated potassium channel slowpoke isoform x7	10.75	8.45E–11	2.06E–08
TR37837-c6_g1_i2	Ae-AP Body	inorganic phosphate cotransporter isoform x1	10.74	6.56E–12	2.22E–09
TR28738-c0_g3_i2	Ae-AP Body	embryonic polarity protein dorsal-like isoform x3	10.66	4.77E–12	1.46E–09
TR20185-c0_g1_i1	Ae-AP Body	transposable element p transposase	10.64	1.09E–11	3.53E–09
TR29865-c1_g2_i1	Ae-AP Body	cordon-bleu 1	10.57	2.74E–10	5.85E–08
TR24635-c2_g2_i3	Ae-AP Body	zinc finger cchc domain-containing protein 4	10.48	6.09E–11	1.16E–08
TR16911-c9_g1_i6	Ae-AP Body	dynein heavy cytoplasmic isoform x2	10.26	2.05E–08	2.66E–06
TR19336-c1_g1_i6	Ae-AP Body	uncharacterized protein LOC100740589 isoform X3	10.18	3.73E–07	3.52E–05
TR41810-c12_g5_i10	Ae-SA Body	vinculin isoform x9	11.09	3.07E–12	1.12E–09
TR42270-c3_g5_i12	Ae-SA Body	calcium-activated potassium channel slowpoke isoform x6	10.99	5.80E–12	1.95E–09
TR21226-c0_g1_i1	Ae-SA Body	nfu1 iron-sulfur cluster scaffold mitochondrial-like	10.94	8.07E–12	2.58E–09
TR19336-c1_g1_i10	Ae-SA Body	fh1 fh2 domain-containing protein 3 isoform x4	10.83	1.69E–11	4.93E–09
TR31615-c2_g1_i1	Ae-SA Body	PREDICTED: uncharacterized protein LOC105456969	10.75	2.70E–11	7.44E–09
TR31615-c7_g1_i1	Ae-SA Body	ubiquitin carboxyl-terminal hydrolase 17-like partial	10.46	1.66E–10	3.67E–08
TR32717-c6_g1_i1	Ae-SA Body	paired amphipathic helix protein sin3a	9.93	3.40E–09	5.60E–07
TR4388-c16_g1_i1	Ae-SA Body	whirlin isoform x1	9.66	1.71E–08	2.32E–06
TR32865-c1_g1_i2	Ae-SA Body	a disintegrin and metalloproteinase with thrombospondin motifs 16 isoform x1	9.64	2.06E–08	2.71E–06
TR13691-c0_g1_i3	Ae-SA Body	membrane metallo-endopeptidase-like partial	9.61	7.62E–13	3.88E–10
TR45167-c1_g2_i2	Ae-SA Body	kielin chordin-like protein isoform x2	9.36	9.44E–08	1.07E–05
TR9096-c9_g1_i6	Ae-SA Body	piezo-type mechanosensitive ion channel component 1 isoform x2	9.28	6.66E–12	2.21E–09
TR49009-c9_g1_i7	Ae-SA Body	voltage-dependent calcium channel subunit alpha-2 delta-3 isoform x2	9.26	1.66E–07	1.76E–05
TR47041-c0_g1_i4	Ae-SA Body	PREDICTED: uncharacterized protein LOC106789540 isoform X2	9.21	2.10E–07	2.14E–05
TR27559-c2_g1_i4	Ae-SA Body	rho gtpase-activating protein 190 isoform x1	9.02	6.48E–07	5.69E–05
TR23225-c0_g2_i6	Ae-SA Body	serine threonine-protein kinase ick-like isoform x2	8.85	1.49E–06	1.17E–04
TR37577-c0_g1_i2	Ae-SA Body	probable 28s rrna (cytosine-c )-methyltransferase	8.79	2.00E–06	1.52E–04
TR48980-c1_g2_i1	Ae-SA Body	creb-binding protein isoform x5	8.77	2.55E–10	4.86E–08
TR45475-c0_g2_i2	Ae-SA Body	ras-related protein m-ras-like	8.61	5.06E–06	3.38E–04
TR34000-c0_g1_i1	Ae-SA Body	neprilysin-2 isoform x1	8.56	9.29E–10	1.50E–07
TR3953-c4_g1_i15	Ae-AP Head	sorbin and sh3 domain-containing protein 1 isoform x3	14.77	5.90E–23	3.41E–19
TR27559-c2_g1_i1	Ae-AP Head	rho gtpase-activating protein 190 isoform x1	14.07	6.39E–22	1.19E–18
TR42270-c3_g5_i10	Ae-AP Head	calcium-activated potassium channel slowpoke	14.04	8.41E–21	1.52E–17
TR16911-c9_g1_i6	Ae-AP Head	dynein heavy cytoplasmic isoform x2	13.97	1.95E–20	2.81E–17
TR27544-c9_g3_i1	Ae-AP Head	bromodomain adjacent to zinc finger domain protein 2b-like isoform x7	13.31	3.90E–18	2.42E–15
TR41810-c12_g5_i6	Ae-AP Head	vinculin isoform x5	12.85	1.59E–18	1.10E–15
TR4006-c7_g2_i9	Ae-AP Head	down syndrome cell adhesion molecule-like protein dscam2 isoform x30	12.76	2.79E–18	1.77E–15
TR36885-c0_g2_i1	Ae-AP Head	oxidoreductase glyr1 homolog	12.74	1.20E–17	6.36E–15
TR52610-c0_g1_i1	Ae-AP Head	sodium hydrogen exchanger 7 isoform x4	12.7	2.18E–17	8.18E–15
TR9088-c5_g1_i4	Ae-AP Head	heterogeneous nuclear ribonucleoprotein k	12.16	1.66E–16	6.35E–14
TR52097-c0_g1_i3	Ae-AP Head	synaptojanin-1 isoform x1	12.07	1.21E–15	3.58E–13
TR53811-c0_g2_i6	Ae-AP Head	e3 ubiquitin-protein ligase hectd1 isoform x4	11.76	3.20E–15	8.91E–13
TR23225-c0_g2_i1	Ae-AP Head	serine threonine-protein kinase ick-like isoform x1	11.75	2.83E–15	7.29E–13
TR8499-c8_g1_i3	Ae-AP Head	netrin receptor unc5c	11.64	7.35E–12	7.20E–10
TR13228-c0_g1_i1	Ae-AP Head	proteasome subunit alpha type-3	11.63	4.91E–14	1.03E–11
TR45093-c0_g1_i2	Ae-AP Head	adamts-like protein 4 isoform x2	11.47	3.37E–14	5.61E–12
TR11878-c8_g1_i2	Ae-AP Head	e3 ubiquitin-protein ligase nedd-4 isoform x1	11.03	1.05E–10	7.80E–09
TR45475-c0_g2_i1	Ae-AP Head	ras-related protein m-ras-like	11	3.97E–13	6.57E–11
TR16826-c0_g1_i1	Ae-AP Head	prenylcysteine oxidase-like	10.48	9.78E–11	1.26E–08
TR53868-c5_g2_i3	Ae-AP Head	caax prenyl protease 1 homolog	10.37	4.92E–08	3.92E–06
TR53728-c7_g1_i11	Ae-SA Head	sodium channel protein para isoform x10	13.95	4.98E–21	6.31E–18
TR49009-c9_g1_i5	Ae-SA Head	voltage-dependent calcium channel subunit alpha-2 delta-3 isoform x2	13.57	6.80E–20	6.57E–17
TR37911-c0_g2_i7	Ae-SA Head	focal adhesion kinase 1 isoform x1	12.6	4.95E–17	1.83E–14
TR43558-c0_g1_i1	Ae-SA Head	PREDICTED: uncharacterized protein LOC107045241	12.54	7.85E–17	2.77E–14
TR9014-c15_g1_i2	Ae-SA Head	a disintegrin and metalloproteinase with thrombospondin motifs 8 isoform x8	12.22	6.79E–16	1.88E–13
TR49009-c9_g1_i4	Ae-SA Head	voltage-dependent calcium channel subunit alpha-2 delta-3 isoform x1	12.01	2.64E–15	6.35E–13
TR31615-c2_g1_i1	Ae-SA Head	PREDICTED: uncharacterized protein LOC105456969	11.92	5.07E–15	1.12E–12
TR27559-c2_g1_i4	Ae-SA Head	rho gtpase-activating protein 190 isoform x1	11.36	3.46E–14	6.76E–12
TR23225-c0_g2_i6	Ae-SA Head	serine threonine-protein kinase ick-like isoform x2	11.24	7.57E–14	1.37E–11
TR42270-c3_g5_i15	Ae-SA Head	calcium-activated potassium channel slowpoke isoform x16	11.15	1.37E–13	2.39E–11
TR28916-c0_g3_i4	Ae-SA Head	liprin-beta-1 isoform x4	11.15	1.39E–13	2.42E–11
TR5686-c0_g1_i1	Ae-SA Head	RNA-directed DNA polymerase from mobile element jockey-like	11.09	2.08E–13	3.48E–11
TR4388-c16_g1_i1	Ae-SA Head	whirlin isoform x1	11.05	2.58E–13	4.25E–11
TR52871-c3_g1_i2	Ae-SA Head	carotenoid isomerooxygenase-like	10.91	6.65E–13	1.02E–10
TR5656-c4_g1_i1	Ae-SA Head	nuclear protein localization protein 4 homolog isoform x2	10.66	3.34E–12	4.52E–10
TR19336-c1_g1_i10	Ae-SA Head	fh1 fh2 domain-containing protein 3 isoform x4	10.26	4.36E–11	4.98E–09
TR41810-c12_g5_i10	Ae-SA Head	vinculin isoform x9	9.99	1.02E–14	2.40E–12
TR30823-c0_g1_i1	Ae-SA Head	zinc finger protein 853-like isoform x1	9.93	3.34E–10	3.24E–08
TR30823-c0_g1_i9	Ae-SA Head	zinc finger protein rotund isoform x7	9.86	5.27E–10	4.90E–08
TR53880-c6_g1_i1	Ae-SA Head	elks rab6-interacting cast family member 1 isoform x2	9.76	1.87E–07	1.27E–05

### Identification of putative chemosensory and olfaction-related genes

Olfaction plays a crucial role in insect behavior such as mate recognition, foraging, host location and host discrimination or finding shelter in complex environments (Suh, Bohbot & Zwiebel, 2014). Behavioral differences in host preference and host acceptance based on aphid host species and plants have been reported previously for *A. ervi* (Zepeda-Paulo et al., 2013). This ability to differentiate between hosts almost certainly involves chemical signal perception (Takemoto, Kainoh & Takabayashi, 2011). As olfactory behavior differences depend on both odorant recognition and signal propagation/processing, genes coding for odorant perception such as Odorant Binding Proteins (OBPs), Chemosensory Proteins (CSPs) and Odorant Receptors (ORs) and genes coding for signal propagation such as voltage-gated sodium channels are prime candidate genes underlying the observed differences in host-preference in *A. ervi* (Zepeda-Paulo et al., 2013). This variation in the ability to perceive and respond to chemosensory cues would provide a target for adaptive evolution (Arya et al., 2015) or phenotypic plasticity. The identification of genes potentially involved in olfactory behavior in *A. ervi* and the highlighted gene expression differences between wasp populations exploiting different aphid lineages provide the first clues to understand the molecular basis of host-fidelity (Li et al., 2013; Zepeda-Paulo et al., 2013; Sepúlveda et al., 2017b). Homology analyses using NR database identified 91 contigs belonging to gene families involved in insect chemoperception such as OBPs (10 transcripts), CSPs (2 transcripts), SNMPs (1 transcript), ORs (76 transcripts, including the conserved odorant co-receptor, Orco) and ionotropic receptors (IRs; 2 transcripts). Compared to other parasitoids in which these gene families had been annotated using genomic approaches or transcriptomic sequencing from antennal tissues, we found less ORs (76 ORs, 10 OBPs, 2 CSPs, 1 SNMP) compared to *Cotesia chilonis* (117 ORs, 8 OBPs, 2 CSPs, 3 SNMPs) (Qi et al., 2015), but more than *Sclerodermus sp.* (8 ORs, 10 OBPs, 10 CSPs, 2 SNMPs) (Zhou et al., 2015). However, these numbers have to be taken with caution because these gene families are extremely difficult to annotate automatically. We found several significantly differentially expressed genes involved in chemical perception between our three *A. ervi* populations, two OBPs and five ORs were expressed at a higher level in the Ae-AP host-race while only one OBP had a higher expression value on the Ae-SA host-race (Table 3). Additionally, we found higher expression of an IR (the glutamate receptor kainate 2) in the Ae-AP host-race relative to Ae-SA host-race (Table 3). Interestingly, increased expression levels of glutamate receptor kainate 2 have been linked to olfactory responses in the salmon louse *Caligus rogercresseyi* (Núñez Acuña et al., 2014).

**Table 3:** Expression patterns of transcripts involved in chemoperception between *Aphidius ervi* populations reared on their natal hosts.

Putative annotation	Log2-fold change	FDR-adjusted *p*-value	Higher in
Odorant Receptor 13a like	4.13	2.26E–003	Ae –AP
Odorant Receptor 20	8.03	1.45E–005	Ae–AP
Odorant Receptor 98	3.24	3.37E–003	Ae–AP
Odorant Receptor OR2-like	8.37	3.22E–004	Ae–AP
Odorant Receptor 13 –isoform x2	3.76	5.98E–005	Ae–AP
Odorant Binding protein 83	4.40	3.78E–004	Ae–AP
Odorant Binding protein 69	3.36	2.34E–004	Ae–AP
Glutamate receptor kainate 2	3.75	2.10E–003	Ae–AP
Odorant Binding protein 56d like	7.63	2.10E–003	Ae–SA

### Cellular signaling and neural development

Within the *A. ervi* reference transcriptome, genes coding for proteins participating in neuronal development and synaptic function of the nervous system were also found, which include the Rho family of GTPases. As Rho signaling activity plays a key role in neural morphological plasticity through dendritic reorganization and structural remodeling of synapses (Tolias, Duman & Um, 2011), it has been linked to long-term memory formation, including olfactory learning (Dobrin & Fahrbach, 2012). Additionally, variation in olfactorybehavior showed by different lines of *Drosophila melanogaster* has been linked to variants in genes involved in nervous systems development and function, such as Rho signaling (Arya et al., 2015).

Remarkably, we found a higher expression for Rho GTPase activating protein 190 (Rho-GAP 190) (Table 2) and Rho guanine nucleotide exchange factor 7 (Rho-GEF 7) (File S2), in the heads of Ae-AP compared to Ae-SA heads. We also found a higher expression for a gene coding for a sodium channel (sodium channel protein 60e) in Ae-AP heads compared to Ae-SA heads (File S1). In *D. melanogaster*, this sodium channel participates in processing olfactory information and regulates olfactory acuity, so its reduced expression impairs olfaction (Kulkarni et al., 2002; Zhang et al., 2013). Furthermore, this sodium channel in *D. melanogaster* is particularly expressed in olfactory organs (third antennal segment and maxillary palps) and brains (Gosselin-Badaroudine et al., 2016; Kulkarni et al., 2002). Other protein involved in cellular signaling is Calmodulin (CaM), a highly-conserved protein that contains four EF-hand domains that allow binding Ca^+2^ ions (Park et al., 2008). Conformational changes in CaM allow its interaction with several target proteins including Orco (through Orco’s CaM binding motif) (Bahk & Jones, 2016) and modulate insect OR function (Mukunda et al., 2014); repetitive subthreshold odor stimulation of olfactory neurons sensitizes ORs, and inhibition of CaM expression abolishes sensitization (Mukunda et al., 2016). Higher expression for a transcript coding for a CaM protein was found in Ae-AP heads compared to Ae-SA heads (neo-calmodulin-like isoform x4; 4.76 logFC; File S1).

## Discussion

Our study provides the first comprehensive reference transcriptome for the aphid parasitoid wasp *Aphidius ervi* obtained from females reared on three different aphid hosts: two host-races of the pea aphid *A. pisum* and the grain aphid *S. avenae*. From the RNA sequencing of head and body of these parasitoid lines, we were able to compare expression profiles and identify putative genes involved in host fidelity (Sepúlveda et al., 2017b). Enriched GO terms in a set of significantly up-regulated genes, suggest possible gene regulatory networks responsible for the observed differences at the phenotypic level. Not surprisingly, a comparison between head and body (thorax/gaster) tissues revealed that the activity related with stimulus perception and processing (signal transduction, transmembrane transport activity among others; Fig. 4.) are highly enriched in head tissue. On the other hand, GO terms enriched in bodies such as peptidase activity (i.e Neprilysin) are probably associated with the presence of transcripts coding for venom proteins, as *A. ervi* females have glands for venom production that is injected at oviposition and enriched in peptidases (Colinet et al., 2014; Falabella et al., 2007; Nguyen et al., 2013).

Behavioral experiments using the same laboratory populations from which the individuals of our transcriptome experiment were taken, showed that *A. ervi* collected from different aphid species (*A. pisum* and *S. avenae*) and host-races of the pea aphids (pea versus alfalfa) differed in several infectivity traits (host preference) but not in virulence (fitness) (Zepeda-Paulo et al., 2013). This is despite the lack of any detectable genetic structuring in Chilean *A. ervi* natural populations collected from different aphid hosts species in the field (Daza-Bustamante et al., 2002; Zepeda-Paulo et al., 2015). The absence of genetic differentiation is explained by the very recent (1970ies) and single introduction event of *A. ervi* as a biological control agent for aphid pests in Chile (Zepeda-Paulo et al., 2016; Zúñiga et al., 1986). The proximal mechanisms of *A. ervi* infectivity (locating, searching and accepting an aphid host) most likely involve chemical cues from either the host-plant complex such as blends of host-induced volatiles produced by parasitized plants (Sasso et al., 2007; Takemoto & Takabayashi, 2015) or directly from the host (e.g., cuticle, cornicle secretions, faeces, exuviae, sex pheromones) (Powell & Wright, 1988). These cues are most likely learned during parasitoid development inside the host (Henry, Roitberg & Gillespie, 2008), during parasitoid emergency (Gutiérrez-Ibáñez, Villagra & Niemeyer, 2007) or imprinted rather than based on genotyping differences in preference, i.e., innate (Antolin, Bjorksten & Vaughn, 2006). Although the influence of possible genotypic variation between *A. ervi* populations can currently not be ruled out, as there could be a genetic basis accounting for variation in olfactory behavior as is the case of *D.  melanogaster* (Arya et al., 2015), further studies need to be carried out to determine whether variations in olfactory behavior are inherited in *A. ervi* or they are just learned based on phenotypic plasticity and differential gene expression.

We know from many examples that the exposure of insects to different environments during juvenile development can lead to substantial differences in the transcriptome of adult individuals (Berens, Hunt & Toth, 2015; Schrader et al., 2015). Hence, in our comparison between head transcriptomes of *A. ervi* reared on different aphid species we focused on differential expression of key components of olfactory and learning pathways (e.g., peripheral system: olfactory receptors, odorant binding proteins, ionotropic receptors, gustatory receptors; nervous system function and development). We identified *in silico* a total of 91 unigenes possessing high-sequence identities with chemosensation-related genes, including IRs, ORs, OBPs, CSPs, SNMPs and Orco, which is similar to what has been reported for other parasitoids species (Qi et al., 2015; Zhou et al., 2015), so our approach proved to be effective for detecting and annotating genes coding for proteins associated with olfaction. As changes in olfactory sensitivity could be driven by mutations in key genes, gene gains and losses, and/or variation in gene expression (Glaser et al., 2015), and due to the lack of genetic differences between parasitoids coming from different hosts, we focused our analysis on gene expression. We found 3 OBPs and 5 ORs that were differentially expressed between head samples of Ae-SA and Ae-AP. Interestingly, both Ae-AP populations (APA and APP) had a higher number of up-regulated chemosensory genes and neuronal-related genes compared to Ae-SA (Table 3–additional file 1), which is similar to that observed in other insect species capable of using different hosts, such as in the Mediterranean corn borer *Sesamia nonagrioides* (Glaser et al., 2015).

We also studied the expression profiles for genes involved in both cellular signaling and neural development. Surprisingly, genes coding for proteins involved in neuronal morphology re-modelling (Tolias, Duman & Um, 2011) had higher transcriptional levels in Ae-AP heads, including Rho-GTPase activating proteins and Rho guanine nucleotide exchange factor, both of which have been described as regulators of Rho signaling activity and have been linked to olfactory learning and long-term memory formation in *A. mellifera* (Dobrin & Fahrbach, 2012), while variants in genes involved in neural development and signaling (e.g., Rho proteins) are related to variation in olfactory behavior in *D. melanogaster* (Arya et al., 2015). Regarding signal transduction, we found higher expression levels in Ae-AP heads for both the sodium channel protein 60e, which participates in processing olfactory information and olfactory acuity (Kulkarni et al., 2002; Zhang et al., 2013), and Calmodulin (CaM), which interacts with Orco through binding of Ca^+2^ ions and modulates insect OR function (Mukunda et al., 2014).

Taken together, our transcriptional evidence coincides with the observation that *A. ervi*—AP shows a higher discrimination in terms of host preference than *A. ervi*—SA (Zepeda-Paulo et al., 2013). This differential expression of candidate chemosensory genes, signaling genes and neuronal development genes may explain host-fidelity in *A. ervi*, and could be a signature of adaptive phenotypic plasticity to different host and host-plant induced environments (Glaser et al., 2015). The genes emphasized in this study deserve special attention for future research in order to prove their role in host preference and host selection by *A. ervi* (i.e, host fidelity). Further studies should also consider detailed analysis both at gene sequence level between *A. ervi* populations, and alternative splicing of these coding genes, as in the case of the tarnished plant bug (*Lygus lineolaris*), where alternative splicing may contribute to the divergence of OBPs (Oppenheim et al., 2015).

More generally, our results provide some input for discussion on the impact of phenotypic plasticity on evolutionary changes. It is indeed puzzling that, although the population does not seem to be subdivided but rather genetically homogenous (most likely due to the relative recent introduction of a limited number of individuals into Chile), we can observe distinct and persistent phenotypic and transcriptional differences between parasitoids coming from different aphid host species and host races. It has been put forward that a developmental reorganization from ancestral phenotypes due to new environmental input or conditions (host races or host biotypes in this case) does not necessarily require new mutations to produce novel or distinct phenotypes (West-Eberhard, 2003). The *A. ervi* populations in Chile may provide an example for developmental reorganization because it seems unlikely that they evolved novel adaptive mutations, as this biocontrol agent was intentionally introduced in few numbers in Chile just about 40 years ago. The next evolutionary step towards speciation based on host races would/could be genetic accommodation, i.e., developmental variants (in our case host races). When novel genetic variants are fixed within populations, those variants could limit phenotypic plasticity, so individuals can no longer easily switch between different phenotypes due to trade-offs and constraints (i.e., reduced fitness if they are not reared on their preferred hosts). It would be interesting to see whether and how many of the differentially expressed genes underlying the observed host related differences in *A. ervi* also show fixed genetic differences.

##  Supplemental Information

10.7717/peerj.3640/supp-1 Table S1 Complete lists of differentially expressed genes for *Aphidius ervi*

10.7717/peerj.3640/supp-2 Supplemental Information 1 Supplementary File Table S2: Final number of reads from each library used in DE analysis.
Figure S1. Sample correlation matrix heatmap for all *Aphidius ervi* libraries.

10.7717/peerj.3640/supp-3 Supplemental Information 2 *Aphidius ervi* filtered transcriptome assembly

## Additional Information and Declarations

### DNA Deposition

The following information was supplied regarding the deposition of DNA sequences: Transcriptomic assembly is available with GenBank accession GFLW01000000. Raw data is available under BioProject ID: PRJNA377544. Filtered transcriptomic assembly is available at: Ballesteros, Gabriel (2017): *Aphidius ervi, de novo* transcriptome assembly, reduced set. figshare.
https://doi.org/10.6084/m9.figshare.4816939.v2.

### Data Availability

The following information was supplied regarding data availability: Raw transcriptomic data is available under BioProject ID: PRJNA377544. Table S1 is available at Ballesteros, Gabriel (2017): differentially expressed gene lists for *A. ervi*. figshare.
https://doi.org/10.6084/m9.figshare.4816942.v4. Table S2 and Fig. S1 are available at: Ballesteros, Gabriel (2017): Read count table mapped and sample correlation matrix—*A. ervi* transcriptome study. figshare.
https://doi.org/10.6084/m9.figshare.5116270.v1. Fasta file of filtered transcriptome assembly is available at: Ballesteros, Gabriel (2017): *Aphidius ervi, de novo* transcriptome assembly, reduced set. figshare.
https://doi.org/10.6084/m9.figshare.4816939.v2. Annotation of transcriptome is available at: Ballesteros, Gabriel (2017): Annotation table for *A. ervi* transcriptome, filtered dataset. figshare.
https://doi.org/10.6084/m9.figshare.4822069.v1.
