# Supplementary material for: Expression differences in Aphidius ervi (Hymenoptera: Braconidae) females reared on different aphid host species
Source: PeerJ. 2017 Aug 21;5:e3640. doi: 10.7717/peerj.3640 (PMC5572533; doi:10.7717/peerj.3640)
Supplement: Supplemental Information 1 — Table S2: Final number of reads from each library used in DE analysis. Figure S1. Sample correlation matrix heatmap for all Aphidius ervi libraries. [file peerj-05-3640-s002.docx]

| **Population** | **Body** | **Head** |
| --- | --- | --- |
| A. ervi - APA | 43,733,854 | 38,876,038 |
| A. ervi - APP | 40,189,908 | 38,403,840 |
| A. ervi - SA | 66,280,364 | 53,068,010 |

**Supplementary Table 2**: Final number of reads from each library used in DE analysis.

**
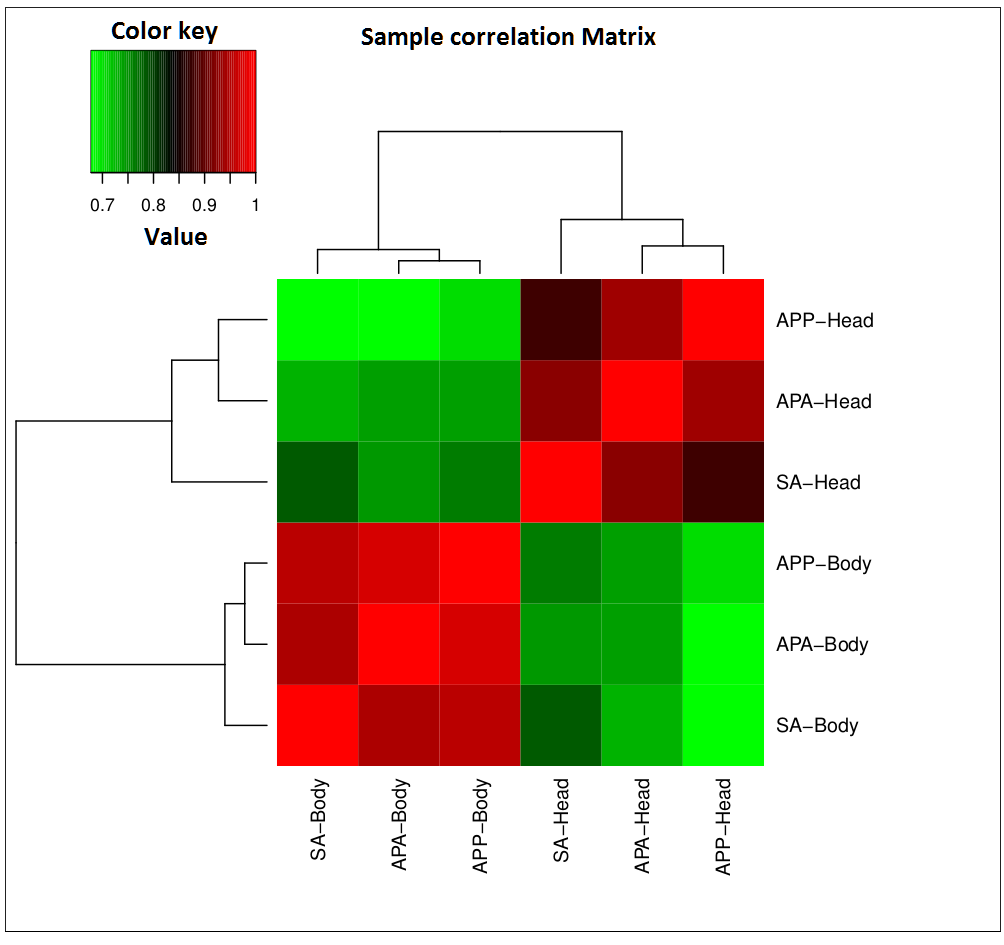
Supplementary Fig 1**. Sample correlation matrix heatmap for all *A. ervi* libraries.
